# Early-Onset Vogt-Koyanagi-Harada-Like Syndrome After Combined Ipilimumab and Nivolumab for Metastatic Melanoma: A Case Report

**DOI:** 10.7759/cureus.113394

**Published:** 2026-07-26

**Authors:** Floriane Etheve, Ewa Hainaut, Damien Boutin, Rodolphe Riviere

**Affiliations:** 1 Dermatology, Poitiers University Hospital, Poitiers, FRA

**Keywords:** immune checkpoint inhibitors, immune-related adverse events, ipilimumab, lichenoid dermatitis, metastatic melanoma, nivolumab, uveitis, vogt–koyanagi–harada-like syndrome

## Abstract

Vogt-Koyanagi-Harada (VKH)-like syndrome is a rare immune-related adverse event associated with immune checkpoint inhibitors (ICIs), resulting from an autoimmune response directed against melanocyte-associated antigens. We report the case of a 56-year-old woman with stage IV metastatic melanoma who developed fever, diffuse pruritic lichenoid dermatitis, and acute bilateral visual loss one week after one cycle of combined ipilimumab and nivolumab. Ophthalmological evaluation demonstrated bilateral serous retinal detachments consistent with VKH-like syndrome, while cerebrospinal fluid analysis confirmed aseptic lymphocytic meningitis. Skin biopsy revealed lichenoid interface dermatitis associated with a mild reduction in melanocyte density demonstrated by Melan-A and SOX10 immunostaining. Immunotherapy was discontinued, and treatment with high-dose systemic corticosteroids followed by methotrexate resulted in rapid clinical improvement. More than two and a half years after permanent discontinuation of ICI therapy, the patient remains in sustained complete radiological remission without resumption of immunotherapy. This case illustrates an uncommon presentation of early-onset VKH-like syndrome following dual immune checkpoint blockade, combining ophthalmologic, neurological, and histologically documented cutaneous involvement. It also highlights the importance of early multidisciplinary management to preserve visual function while optimizing oncological care. Although VKH-like syndrome cannot currently be considered a validated predictive biomarker, accumulating evidence suggests that melanocyte-directed autoimmunity may reflect a particularly effective antitumor immune response in a subset of patients with metastatic melanoma.

## Introduction

Vogt-Koyanagi-Harada (VKH)-like syndrome is a rare immune-related adverse event associated with immune checkpoint inhibitors (ICIs), characterized by bilateral granulomatous panuveitis associated with variable neurological, auditory, and cutaneous manifestations. Its pathogenesis is thought to involve a T-cell-mediated immune response directed against melanocyte-associated antigens in genetically predisposed individuals, particularly carriers of HLA-DRB1*04 alleles [1-5]. Although classical VKH is an idiopathic autoimmune disorder, VKH-like syndrome has become an increasingly recognized complication of ICIs, particularly in patients with metastatic melanoma, likely because melanoma cells and normal melanocytes share differentiation antigens targeted by activated cytotoxic T lymphocytes [4,5]. Early recognition and prompt initiation of systemic corticosteroid therapy are essential to preserve visual function and reduce the risk of chronic ocular complications [1,3,6].

ICIs have dramatically improved survival in patients with advanced melanoma but may induce immune-related adverse events involving virtually every organ system. Ocular toxicities remain uncommon, with VKH-like syndrome representing one of the rarest ophthalmologic immune-related adverse events. A recent systematic review identified 52 published cases of ICI-associated VKH-like syndrome, underscoring both the rarity of this condition and the importance of early multidisciplinary diagnosis and management [7]. A recent review further highlighted the value of multimodal ocular imaging and emphasized the need for close collaboration between oncologists and ophthalmologists to optimize diagnosis and treatment [8].

We report the case of a 56-year-old woman who developed early-onset VKH-like syndrome with bilateral serous retinal detachments, aseptic lymphocytic meningitis, and histologically confirmed lichenoid dermatitis shortly after one cycle of combined ipilimumab and nivolumab for metastatic melanoma. Despite permanent discontinuation of immunotherapy, she experienced rapid clinical improvement following systemic corticosteroids and methotrexate and remains in sustained complete radiological remission more than two and a half years after ICI discontinuation without resumption of immunotherapy. This case expands the clinical spectrum of ICI-associated VKH-like syndrome and highlights the importance of early multidisciplinary recognition and management of this rare immune-related adverse event.

## Case presentation

A 56-year-old woman with no history of autoimmune disease was diagnosed with stage IV BRAF V600-mutant cutaneous melanoma arising from the right lumbar region (Breslow thickness, 3.25 mm), with regional and distant lymph node metastases. First-line treatment with combined ipilimumab and nivolumab was initiated.

Three days after the first infusion, she developed persistent fever reaching 39°C that lasted approximately 48 hours. One week after treatment initiation, she presented with an intensely pruritic, symmetrical erythematous maculopapular eruption involving both lower extremities, which rapidly spread to the back, neck, and upper chest.

**Figure 1:**
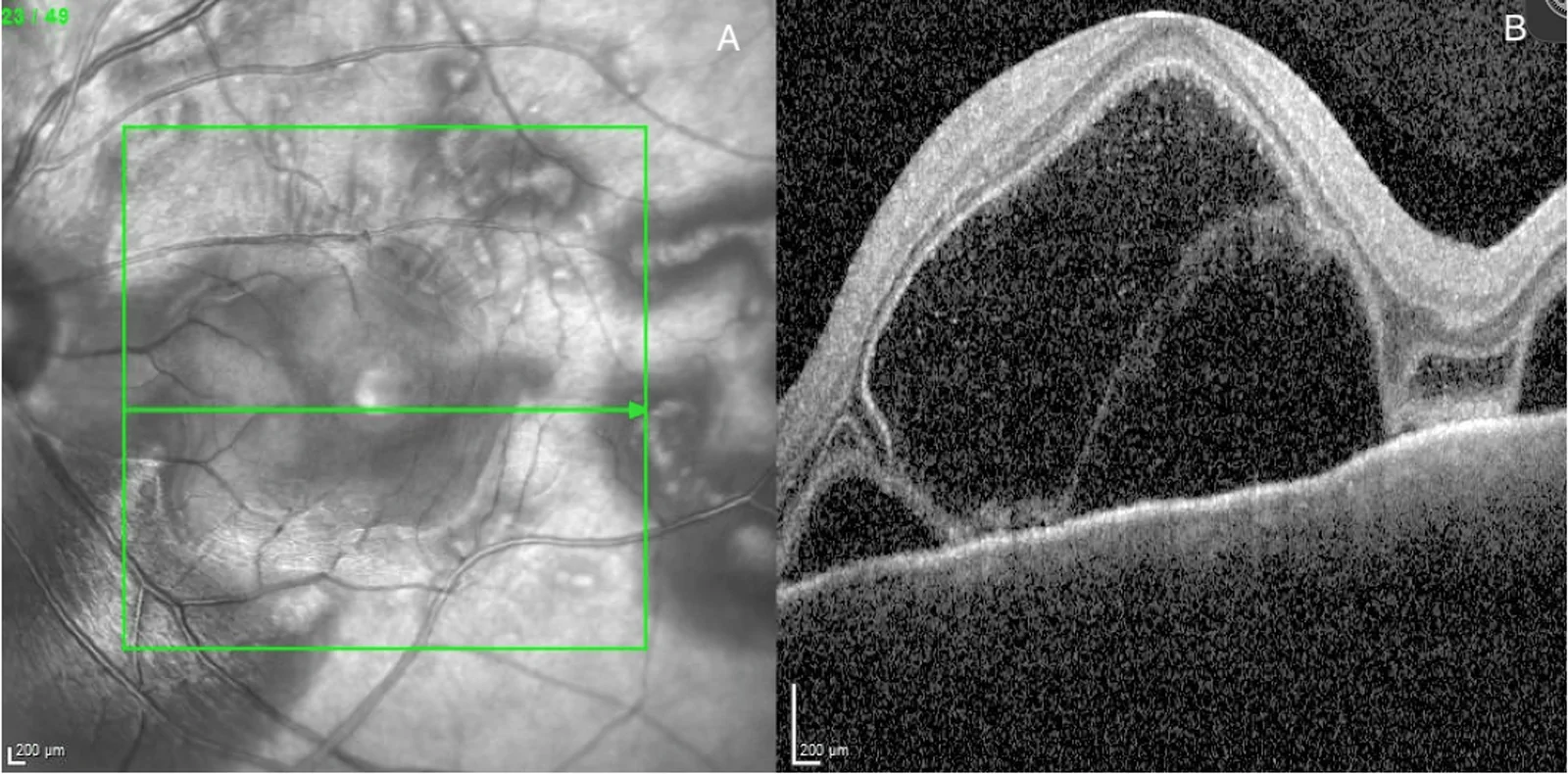
Ophthalmological findings at presentation. Infrared fundus image (A) showing the location of the OCT scan. Spectral-domain optical coherence tomography (B) demonstrates multiple serous neurosensory retinal detachments associated with retinal pigment epithelium detachments, findings consistent with acute Vogt–Koyanagi–Harada (VKH)-like syndrome.

Ten days after the first infusion, she experienced an abrupt bilateral decrease in visual acuity. Ophthalmologic examination revealed bilateral serous retinal detachments associated with retinal pigment epithelium detachments, highly suggestive of Vogt-Koyanagi-Harada (VKH)-like syndrome (Figure 1).

Laboratory investigations are summarized in Table 1.

**Table 1:** Summary of laboratory investigations at the time of diagnosis.

Parameters	Patient values	Reference range
Hepatic cytolysis	Approximately 2 × ULN	
Anicteric cholestasis	Present	Normal
Calcium	2.62 mmol/L	2.15–2.55 mmol/L
Lactate dehydrogenase (LDH)	223 IU/L	<214 IU/L
Thyroid-stimulating hormone (TSH)	0.18 mIU/L	0.4–4.0 mIU/L
Antinuclear antibodies (ANA)	Positive (1:640)	Negative
Anti-double-stranded DNA antibodies	Negative	Negative
Extractable nuclear antigen (ENA) antibodies	Negative	Negative
Rheumatoid factor	Negative	Negative
Anti-cyclic citrullinated peptide (anti-CCP) antibodies	Negative	Negative
Anti-glomerular basement membrane antibodies	Negative	Negative
Cerebrospinal fluid analysis	Aseptic lymphocytic meningitis; no malignant cells	Normal CSF; no malignant cells

They showed mild hepatocellular injury associated with anicteric cholestasis, mild hypercalcemia, elevated lactate dehydrogenase, suppressed thyroid-stimulating hormone, positive antinuclear antibodies (1:640), and otherwise negative autoimmune serologies. Cerebrospinal fluid analysis revealed aseptic lymphocytic meningitis without malignant cells, confirming neurological involvement.

**Figure 2:**
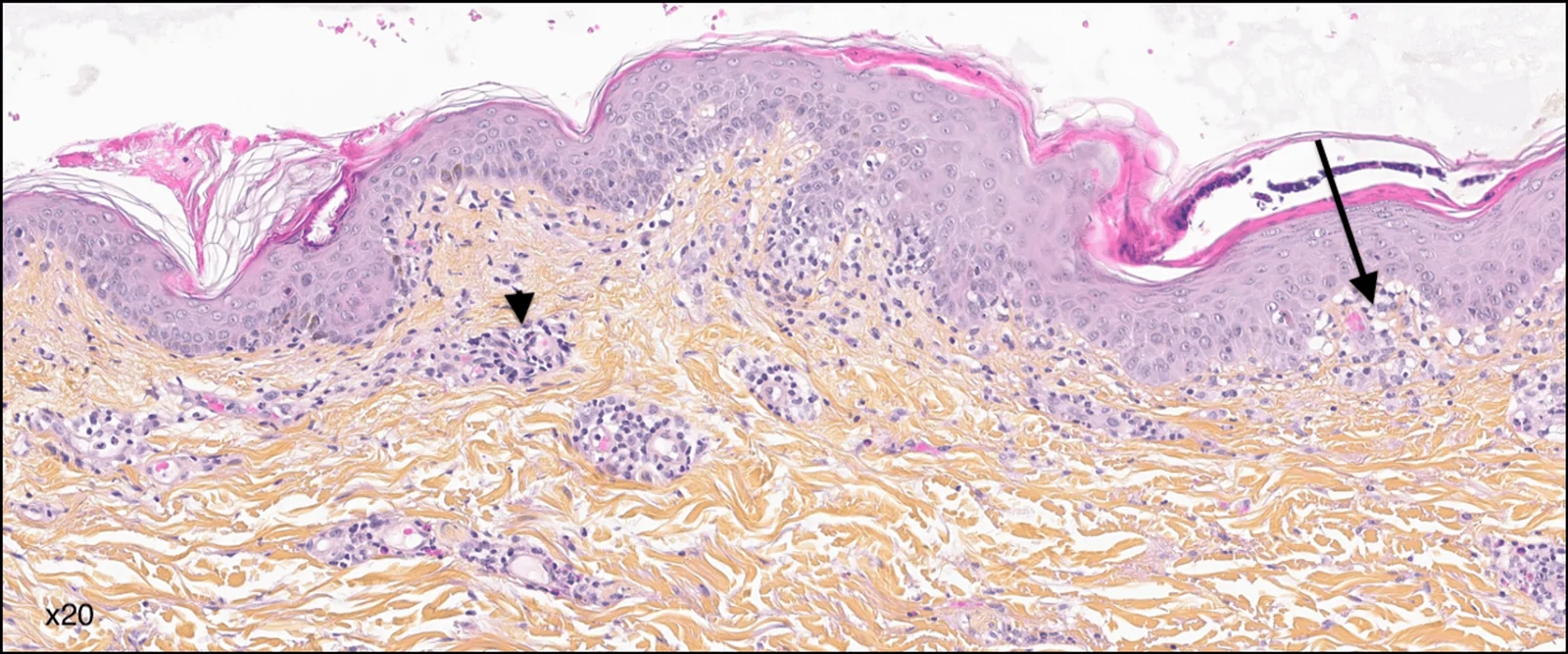
Histopathological findings. Hematoxylin and eosin staining of the skin biopsy showing lichenoid interface dermatitis with basal vacuolar alteration (arrow) and a superficial predominantly lymphocytic perivascular infiltrate (arrowhead). Mild acanthosis is also present. Original magnification ×20.

Skin biopsy demonstrated lichenoid interface dermatitis characterized by mild acanthosis, basal vacuolar degeneration, and a predominantly superficial perivascular lymphocytic infiltrate (Figure 2).

**Figure 3:**
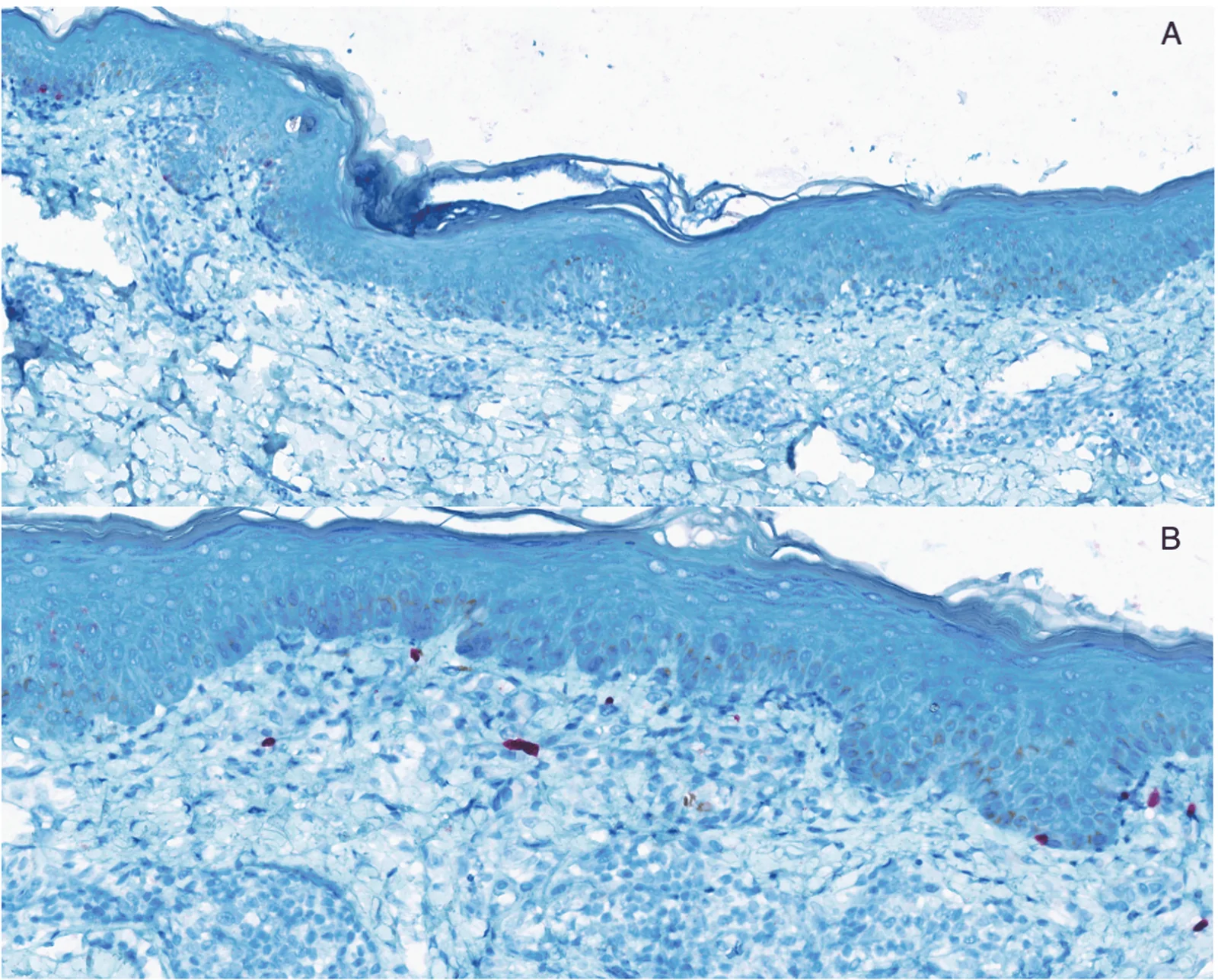
Immunohistochemical findings. (A) Melan-A immunostaining demonstrating a mild reduction in epidermal melanocyte density. (B) SOX10 immunostaining confirming reduced melanocyte density.

Immunohistochemical staining with Melan-A and SOX10 demonstrated a mild reduction in melanocyte density (Figure 3).

These findings were considered compatible with either VKH-like syndrome involving the skin or an ICI-induced lichenoid dermatitis.

Following multidisciplinary discussion involving oncologists, dermatologists, ophthalmologists, and neurologists, a diagnosis of ICI-associated VKH-like syndrome was established based on the combination of characteristic ocular findings and aseptic lymphocytic meningitis. No auditory manifestations were identified.

ICI therapy was permanently discontinued after the first cycle. Initial treatment consisted of two subconjunctival corticosteroid injections administered four days apart, followed by oral prednisone initiated at 1 mg/kg/day (60 mg/day) immediately after diagnosis. Prednisone was gradually tapered and discontinued approximately four months later. Methotrexate (17.5 mg/week) was initiated at the same time as a steroid-sparing agent and discontinued approximately eight months later. Ocular, neurological, and cutaneous manifestations improved rapidly after treatment initiation.

Approximately one year after symptom onset, the patient experienced an isolated ocular relapse that resolved with topical dexamethasone eye drops (one drop every hour for 48 hours, followed by one drop eight times daily for 10 days), atropine eye drops (one drop twice daily for five days), and dexamethasone/oxytetracycline ophthalmic ointment (once daily at bedtime for 10 days).

At the latest follow-up (approximately two and a half years after ICI discontinuation), ICI therapy has not been resumed. The patient remains in sustained complete radiological remission. Apart from an isolated ocular relapse approximately one year after symptom onset, no further VKH-like manifestations have occurred.

## Discussion

VKH-like syndrome is an uncommon but increasingly recognized ocular immune-related adverse event associated with ICIs, particularly in patients with metastatic melanoma. The underlying mechanism is thought to involve a T-cell-mediated immune response directed against melanocyte-associated antigens shared by melanoma cells and normal melanocytes, resulting in cross-reactive autoimmune inflammation of melanocyte-rich tissues, including the uvea, meninges, skin, and hair follicles [2,4,5]. Prompt recognition and early initiation of systemic corticosteroid therapy remain essential to preserve visual function and prevent chronic ocular complications [1,3,6].

Our patient developed fever, diffuse pruritic lichenoid dermatitis, and acute bilateral visual loss within 10 days after a single cycle of combined ipilimumab and nivolumab. Such an early onset suggests rapid activation of pre-existing autoreactive T-cell clones rather than progressive immune sensitization. In addition to bilateral serous retinal detachments, cerebrospinal fluid analysis demonstrated aseptic lymphocytic meningitis, fulfilling the SUN criteria for incomplete VKH-like syndrome [3]. Although neurological involvement is well recognized in classical VKH disease, cerebrospinal fluid-confirmed aseptic lymphocytic meningitis has been only infrequently reported in ICI-associated VKH-like syndrome, making our observation clinically noteworthy [7,9-13].

The cutaneous manifestations observed in our patient deserve particular attention. Classical VKH typically presents with vitiligo, poliosis, and alopecia during the convalescent phase, whereas our patient developed an early diffuse lichenoid dermatitis. Histopathological examination demonstrated interface dermatitis associated with basal vacuolar degeneration, superficial lymphocytic infiltration, and a mild reduction in melanocyte density demonstrated by Melan-A and SOX10 immunostaining. Although lichenoid dermatitis is a well-recognized cutaneous immune-related adverse event associated with ICIs, its concomitant occurrence with ocular and neurological VKH-like syndrome has only rarely been described. This observation broadens the clinical spectrum of ICI-associated VKH-like syndrome and illustrates the diagnostic challenge of distinguishing VKH-related cutaneous involvement from a concomitant immune-mediated lichenoid eruption [5].

A recent systematic review by Zhang et al. identified 52 patients with ICI-associated VKH-like syndrome, nearly 70% of whom had metastatic melanoma. As the available evidence consists almost exclusively of case reports and small case series, the true incidence of this condition remains unknown. Most patients were treated with systemic corticosteroids, approximately 60% discontinued immunotherapy, and visual recovery was achieved in the majority of cases [7].

A complementary review focusing on ICI-associated uveitis emphasized the importance of multimodal ocular imaging, including optical coherence tomography and fluorescein angiography, together with close collaboration between ophthalmologists and oncologists to ensure early diagnosis and appropriate treatment. The authors also highlighted that decisions regarding continuation or rechallenge of ICIs should be individualized according to the severity of ocular inflammation, visual recovery, and expected oncological benefit, as no standardized management strategy currently exists [8].

Interestingly, favorable oncological outcomes have frequently been reported despite discontinuation of immunotherapy in patients who develop VKH-like syndrome [9-13]. Similar observations have also been described with BRAF and MEK inhibitors, suggesting that melanocyte-directed autoimmunity may accompany an effective antitumor immune response [14-17]. This concept parallels melanoma-associated vitiligo, which has consistently been associated with durable responses and improved survival during immunotherapy [5]. Nevertheless, the available evidence remains limited to case reports and small retrospective series and is therefore subject to publication bias. Consequently, VKH-like syndrome should currently be regarded as a potential marker of robust systemic immune activation rather than a validated predictive biomarker of treatment efficacy.

Our case expands the clinical spectrum of ICI-associated VKH-like syndrome by illustrating an unusually early presentation after a single cycle of combined ipilimumab and nivolumab, associated with neurological involvement and histologically confirmed lichenoid dermatitis. Beyond emphasizing the importance of early multidisciplinary recognition and treatment, this observation supports the hypothesis that melanocyte-directed autoimmunity may reflect a robust antitumor immune response. Further prospective studies and international pharmacovigilance registries are needed to clarify the prognostic significance of VKH-like syndrome and to guide decisions regarding immunotherapy continuation or rechallenge.

## Conclusions

VKH-like syndrome is a rare but potentially vision-threatening immune-related adverse event associated with ICIs. Early recognition and prompt multidisciplinary management involving oncologists, ophthalmologists, dermatologists, and neurologists are essential to preserve visual function while ensuring optimal oncological care.

Our case expands the clinical spectrum of ICI-associated VKH-like syndrome by illustrating an uncommon presentation characterized by very early onset following one cycle of combined ipilimumab and nivolumab, concomitant neurological involvement, and histologically confirmed lichenoid dermatitis with reduced melanocyte density. Despite permanent discontinuation of ICI therapy after a single treatment cycle, the patient experienced rapid clinical improvement following systemic corticosteroids and methotrexate and remains in sustained complete radiological remission more than two and a half years after ICI discontinuation without resumption of immunotherapy.

Although VKH-like syndrome should not currently be considered a validated predictive biomarker, accumulating evidence suggests that melanocyte-directed autoimmunity may reflect a robust antitumor immune response in a subset of patients with metastatic melanoma. Further prospective studies and international pharmacovigilance registries are warranted to better define its prognostic significance and to establish evidence-based recommendations regarding immunotherapy continuation or rechallenge following resolution of this rare but clinically significant immune-related adverse event.
